# Peripheral Arterial Stiffness Is Independently Associated with a Rapid Decline in Estimated Glomerular Filtration Rate in Patients with Type 2 Diabetes

**DOI:** 10.1155/2013/309294

**Published:** 2013-12-29

**Authors:** Yi-Jing Sheen, Jiann-Liang Lin, Tsai-Chung Li, Cho-Tsan Bau, Wayne Huey-Herng Sheu

**Affiliations:** ^1^Division of Endocrinology and Metabolism, Department of Internal Medicine, Taichung Hospital, Department of Health, Executive Yuan, No. 199, Section 1, Sanmin Road, Taichung 402, Taiwan; ^2^Institute of Biostatistics, China Medical University, No. 91 Hsueh-Shih Road, Taichung 404, Taiwan; ^3^Division of Endocrinology and Metabolism, Department of Internal Medicine, Taichung Veterans General Hospital, No. 1650, Section 4, Taiwan Boulevard, Taichung 407, Taiwan; ^4^School of Medicine, National Defense Medical Center, Taipei, Taiwan; ^5^School of Medicine, National Yang-Ming University, Taipei, Taiwan; ^6^Institute of Medical Technology, National Chung-Hsing University, Taichung, Taiwan

## Abstract

*Introduction*. Diabetes and its vascular complications are main noncommunicable chronic diseases and major global health issues. Peripheral arterial disease (PAD) is highly prevalent in diabetes with nephropathy. We evaluated the associations of variables of arterial stiffness and the decline in estimated glomerular filtration rate (eGFR) in patients with type 2 diabetes. *Materials and Methods*. A total of 577 type 2 diabetic patients (mean ± SD: age, 63 ± 11 years) were enrolled. A rapid decline in eGFR was defined as progressively lower eGFR detected at both the 6- and 12-month follow-up visits, plus a reduction in eGFR more than 3 mL·min^−1^per 1.73 m^2^ per year. *Results*. Higher glycated hemoglobin (HbA1c), systolic blood pressure (SBP), pulse pressure (PP), and brachial-ankle pulse wave velocity (ba-PWV) at baseline were independently associated with a rapid decline in eGFR. The adjusted odds ratios (95% confidence intervals) for a rapid decline in eGFR for ba-PWV, SBP, and PP were 1.072 (1.011–1.136), 1.014 (1.004–1.025), and 1.025 (1.008–1.041), respectively, after adjustment for gender, age, body mass index, smoking, HbA1c, and baseline eGFR in separated models. *Conclusions*. Ba-PWV may serve as a simple and noninvasive predictor of rapid renal function progression in type 2 diabetic patients.

## 1. Introduction

With rapid growth in patients with type 2 diabetes, a varying degree of renal complications caused by chronic kidney disease (CKD) has imposed a high burden of disease-related morbidity and mortality [1]. In fact, diabetic renal disease has attracted a considerable amount of attention because the prevalence of CKD, end-stage renal disease, and dialysis in patients with diabetes increased from past decades [2]. Although nephropathy is considered a microvascular complication of diabetes, nephropathy shares many risk factors with cardiovascular disease and peripheral artery disease (PAD) [3], such as hypertension [4–6], hyperglycemia [4, 7], dyslipidemia [8, 9], and smoking [7]. Previous studies have shown that, in patients with diabetes, a low estimated glomerular filtration rate (eGFR) is associated with progressive vascular stenosis and stiffening, reflected by worsening values of the ankle-brachial index (ABI), toe-brachial index (TBI), and carotid-femoral pulse wave velocity (cf-PWV) [10–12]. A recent report indicates that cf-PWV is associated with a decline in eGFR in a population of Asian patients with type 2 diabetes [12]. However, measuring cf-PWV is time-consuming and costly and requires a skilled assessor [13, 14]. ABI is a simple, noninvasive, and easy technique for measuring peripheral arterial arthrosclerosis and stiffness. According to the recommendations of the American Diabetes Association [15] and the Clinical Guideline for Diabetes Care in Taiwan [16], ABI should be measured as part of routine screening in all diabetic patients older than 50 years and diabetic patients younger than 50 years with risk factors for PAD, such as smoking or hyperlipidemia. Therefore, we aimed at evaluating risk factors for the prediction of a rapid decline in renal function in patients with type 2 diabetes and at assessing the use of peripheral vascular functional markers for the early prediction of rapid eGFR progression.

## 2. Materials and Methods

### 2.1. Study Population

Data were adapted from a retrospective cohort study conducted at an outpatient clinic in central Taiwan. We enrolled type 2 diabetic patients who regularly visited the outpatient departments of our hospital for at least 1 year and underwent ABI, TBI, and brachial-ankle pulse wave velocity (ba-PWV) examinations between October 2008 and August 2009. In addition, the renal function of all enrolled patients was evaluated at the 6 and 12 months after baseline at follow-up visits. These values were used to derive the slope of the eGFR. In addition, baseline clinical characteristics and measurements of the ABI, TBI, and ba-PWV were collected. Patients were excluded if they were undergoing dialysis or had a history of stroke, stage V chronic kidney disease (eGFR <15 mL·min^−1^per 1.73 m^2^), malignancy, acute myocardial infarction, amputations, or arrhythmia. Those with an ABI of >1.3 or <0.95 were also excluded. An ABI of >1.3 indicates calcification in the lower limbs, and it is difficult to assess for possible obstructive lesions [17], while 0.95 is the ABI cutoff value at which the accuracy of the ba-PWV measurement decreases [18]. Recorded baseline values included body mass index (BMI), systolic blood pressure (SBP), diastolic blood pressure (DBP), and pulse pressure (PP). Biochemical data including fasting plasma glucose, glycated hemoglobin (HbA1c), total cholesterol (TC), low-density lipoprotein cholesterol (LDL), high-density lipoprotein cholesterol (HDL), triglyceride (TG), and serum creatinine were also collected. Calculated by the 4-variable (or abbreviated) Modification of Diet in Renal Disease Study equation: eGFR (mL·min^−1^per 1.73 m^2^) = 186 × Cr^−1.154^  × age^−0.203^ × (0.742, if female) × (1.212, if African American) [19], eGRF was obtained at baseline and at 6 and 12 months. Baseline and 12-month urinary albumin excretion, evaluated by urine albumin-to-creatinine ratio (UACR), were obtained.

### 2.2. Measurement of PAD Indexes (ba-PWV, ABI, and TBI)

ABI, TBI, and ba-PWV were measured using the Vascular Profiler 1000 (Colin Co. Ltd., Komaki, Aichi, Japan), while patients were in the supine position and after resting for at least 5 min in an air-conditioned room (approximately 25°C). SBP, DPP, ba-PWV, and electrocardiogram data were recorded simultaneously. The equation used to calculate ba-PWV is as follows: ba-PWV = (La − Lb)/Tba, where La represents the length from the suprasternal notch to the ankle (La = 0.8129 × height (cm) + 12.328), Lb represents the length from the suprasternal notch to the right brachium (Lb = 0.2195 × height (cm) − 2.0734), and Tba is the time interval between the brachial and ankle arteries measured between the front wave of the brachial and ankle waveforms [20]. ABI was determined as the ratio of ankle SBP to brachial SBP, and TBI was the ratio of toe SBP to brachial SBP. The highest left and right brachial systolic pressures were measured and used to calculate the ABI and TBI automatically. In addition, electrocardiograph electrodes were attached to both wrists, and a microphone for phonocardiography was placed at the third intercostal space on the left margin of the sternum. Pneumatic pressure cuffs were placed snugly around the arms and ankles. Toe blood pressure was measured using a 25 mm wide digital cuff around the base of both first toes. All subjects underwent ba-PWV analysis and bilateral ABI and TBI examinations, and data from the leg with the lower ABI value were analyzed. The same operator performed all examinations during the investigation period; thus, the operator-related variability was low. Validation of these methods was described previously [18].

### 2.3. Clinical and Laboratory Examinations

Clinical data (age, gender, height, and weight) were entered into the profiler. The device measured these data simultaneously. SBP refers to the highest brachial SBP of the patient, and DBP is the brachial DBP measured on the same side as the SBP. PP was calculated as SBP minus DBP. We reviewed patients' medical records to obtain their history of smoking (never, former smoker or current smoker) and medications at baseline. Overnight fasting blood samples were collected for measurement of plasma glucose, HbA1c, and serum cholesterol levels. HbA1c levels were measured by boronate affinity high-performance liquid chromatography (Bio-Rad variant II HbA1C REORDER PACK 1000TEST/PKG, Alfred Nobel Drive Hercules, California, USA). Fasting plasma glucose levels were measured by the glucose oxidase-peroxidase method. Serum cholesterol levels were assayed using the enzymatic method, and triglyceride levels were assayed with the enzymatic GPO-Trinder. The homogeneous method was used to measure high-density lipoprotein cholesterol and low-density lipoprotein cholesterol concentrations, and the UniCel DxC 800 Chemistry Analyzer (Beckman Coulter, Inc., USA) was used to determine serum creatinine concentration. Spot urinary albumin was measured by turbidimetric method and urine creatinine was measured using the modified kinetic Jaffé method (Beckman Coulter, Inc., Fullerton, CA, USA).

### 2.4. Definition of Rapid Decline in Renal Function

The Kidney Disease Outcomes Quality Initiative guidelines developed by the National Kidney Foundation state that eGFR should normally decrease by 1 mL·min^−1^·1.73 m^−2^ per year [21, 22]. A recent study, which included 329 diabetic patients with a mean age of 60 ± 12 years and evaluated the rate of progression of diabetic CKD in different ethnic groups, reported that the annual decline of eGFR was 1.69 mL·min^−1^per 1.73 m^2^ in the overall population and 1.51 mL·min^−1^per 1.73 m^2^ per year in south Asian patients [23]. Based on other previous studies, the definition of a rapid decline in eGFR is an eGFR slope >3 mL·min^−1^per 1.73 m^2^ per year [24, 25]. In the present study, at least 3 eGFR measurements were required to evaluate the eGFR slope. Only patients with progressively lower eGFR values at both the 6- and the 12-month follow-up visits, and plus an eGFR reduction >3 mL·min^−1^per 1.73 m^2^ per year, were defined as having a rapid decline in eGFR; those who did not show such changes were considered to be patients without a rapid decline in eGFR.

### 2.5. Statistical Analyses

All descriptive data are presented as mean ± standard deviation (SD). Statistical analyses were performed using independent sample *t*-tests to detect differences between patients with and without a rapid decline in eGFR. Chi-square tests were used to assess differences based on gender, smoking status, and the use of medications. The adjusted odds ratios (OR) of risk factors for a rapid decline in eGFR were determined by multivariate logistic regression analysis. Receiver operating characteristic curves for ba-PWV, PP, SBP, and TBI levels were constructed, and the area under each curve (AUC) was calculated. Statistical significance was defined as *P* < 0.05. The linear correlations between arterial functional markers at baseline and the change of eGFR were determined by Pearson correlation. All statistical analyses were performed using the Statistical Package for the Social Sciences (SPSS) version 19.0 for Windows (SPSS Inc., Chicago, IL, USA).

## 3. Results

A total of 577 type 2 diabetic patients fulfilled the inclusion criteria with medical records containing at least 3 eGFR measurements at baseline, 6 months, and 12 months. Patients had a mean age of 63 ± 11 years (283 women, 294 men). The mean baseline value of eGFR was 92 ± 28 mL·min^−1^per 1.73 m^2^; 116 patients (20.1%) experienced a rapid decline in eGFR, whereas the remainder (*n* = 461, 79.9%) did not.  Table 1 shows that patients with a rapid decline in eGFR had significantly higher SBP, PP, and ba-PWV values, as well as higher HbA1c levels at baseline (all *P* < 0.05). Although TBI values were lower and the fasting plasma glucose levels were higher in patients with rapid renal function decline, the differences were not significant. The distributions of each medication profile were similar between the 2 groups, except that sulfonylurea was used more commonly in those with rapid eGRF decline (*P* = 0.004) (Table 1). The baseline and 12-month UACR values were obtained in 215 patients (37% of total studied population) with mean age of 61 ± 11 years, male/female ratio: 117/98, and mean eGFR: 94 ± 29 mL·min^−1^per 1.73 m^2^. Those patients with a rapid decline in eGFR (*n* = 52) had significantly higher baseline UACR (377 ± 924 versus 92 ± 275 mg/g, *P* = 0.001, using independent sample *t*-tests) than those without rapid decline of eGFR (*n* = 163). We also detected that higher baseline baPWV levels were associated with progression of UACR during one-year period (according to the values of ba-PWV, patients were separated as three groups: ba-PWV < 14.0 m/s, 1.4–1.8 m/s and ba-PWV > 1.8 m/s; each group has 23%, 38%, and 47% of patients with progression of UACR, resp., analyzed by Chi-square test, *P* = 0.077).

**Figure 1:**
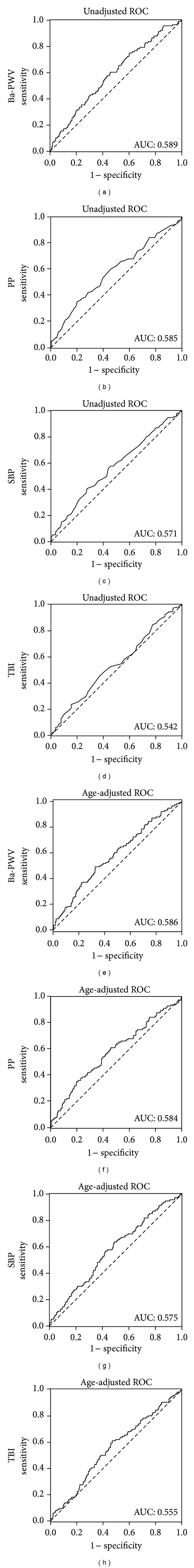
Unadjusted and age-adjusted area under the curve for predicting a rapid decline in estimated glomerular filtration rate according to different vascular functional markers. AUC: areas under the curve, ROC: receiver operating characteristic curves, OR: odds ratio, 95% CI: 95% confidence interval. (a) The unadjusted AUC of ROC curve (95% CI; *P*) for predicting a rapid decline in eGFR for ba-PWV: 0.589 (0.531–0.646; *P* = 0.003*). (b) The unadjusted AUC of ROC curve (95% CI; *P*) for predicting a rapid decline in eGFR for PP: 0.585 (0.525–0.645; *P* = 0.005*). (c) The unadjusted AUC of ROC curve (95% CI; *P*) for predicting a rapid decline in eGFR for SBP: 0.571 (0.512–0.630; *P* = 0.018*). (d) The unadjusted AUC of ROC curve (95% CI; *P*) for predicting a rapid decline in eGFR for TBI: 0.542 (0.484–0.600; *P* = 0.160). (e) The age-adjusted AUC of ROC curve (95% CI; *P*) for predicting a rapid decline in eGFR for ba-PWV: 0.586 (0.528–0.644; *P* = 0.004*). (f) The age-adjusted AUC of ROC curve (95% CI; *P*) for predicting a rapid decline in eGFR for PP: 0.584 (0.524–0.644; *P* = 0.005*). (g) The age-adjusted AUC of ROC curve (95% CI; *P*) for predicting a rapid decline in eGFR for SBP: 0.575 (0.518–0.632; *P* = 0.012*). (h) The age-adjusted AUC of ROC curve (95% CI; *P*) for predicting a rapid decline in eGFR for TBI: 0.555 (0.497–0.613; *P* = 0.067), *P < 0.05.

The effects of aging on eGFR are well known [26]. Therefore, we evaluated all potential risk factors after adjusting for the effect of age. The crude and age-adjusted OR and 95% confidence intervals (95% CI) of baseline parameters are shown in Table 2. Baseline values of HbA1c, SBP, PP, and ba-PWV were significantly greater for patients with a rapid decline of eGFR, after adjustment for age. For the arterial functional markers ba-PWV, PP, SBP, and TBI, the unadjusted and age-adjusted area under the receiver operating curve for predicting a rapid decline in eGFR were 0.589, 0.585, 0.571, and 0.542 and 0.586, 0.584, 0.575, and 0.555, respectively (Figure 1). Apart from TBI, the *P* values for the AUC of these arterial functional markers were all < 0.05, indicating statistical significance (AUC > 0.5) (Figure 1).

Results of the correlation analysis between several arterial functional indexes and eGFR are shown in Table 3. Significant negative correlations between ba-PWV with baseline eGFR, 12-month eGFR, the annual change of eGFR, and the annual change rate of eGFR were observed. In addition, significant negative correlations between SBP with baseline eGFR, 12-month eGFR, the annual change of eGFR, and the annual change rate of eGFR were detected.

Multivariate logistic regression models were built by adding ba-PWV, PP, SBP, and TBI separately after adjusting for potential confounders, including gender, age, body mass index, smoking, HbA1c, and baseline eGFR because of the strong associations between these variables shown in Table 3. The adjusted OR (95% CI; *P*) for a rapid decline in eGFR for ba-PWV, SBP, PP, and TBI were 1.072 (1.011–1.136; *P* = 0.020), 1.014 (1.004–1.025; *P* = 0.009), 1.025 (1.008–1.041; *P* = 0.003), and 0.338 (0.062–1.846; *P* = 0.210), respectively (Table 4(a)). We also established models that were adjusted for the potential influence of sulfonylurea use (independent-sample *t*-test *P* = 0.004) and found that markers of arterial stiffness remained independent risk factors for a rapid decline in eGFR, whereas the effects of HbA1C on rapid renal function progression became nonsignificant (Table 4(b)).

## 4. Discussion

The major findings of the present study were that, in addition to glycemic control (measured by HbA1c) and blood pressure (both SBP and PP), arterial compliance (measured by ba-PWV) was associated with a rapid decline in eGFR during a 1-year follow-up period in type 2 diabetic outpatients without apparent cardiovascular disease or peripheral arterial occlusive disease (ABI values within normal).

### 4.1. Relationship between PAD Indexes, Arterial Stiffness, and eGFR

The presence of arterial stiffness in patients with cardiovascular pathology may be observed even at an early stage of the disease. Previous studies have demonstrated that ba-PWV is a predictor of the future development of coronary artery disease, major cardiovascular events, and heart failure and that it is a factor in the short-term prognosis of type 2 diabetes in patients with coronary artery disease [27, 28]. In addition, ba-PWV predicted aortic-complicated lesions in stroke patients [29], and methods for evaluating arterial stiffness under ambulatory conditions are emerging [30]. A recent study demonstrated that aortic stiffness, measured by cf-PWV, was associated with decreased eGFR in a group of 461 Japanese patients with type 2 diabetes who were followed for a median period of 5.9 years [12]. Another study, which evaluated patients with never-treated hypertension who had normal or minimally reduced eGFR, reported that reduced eGFR is independently associated with increased arterial stiffness, measured by heart-femoral pulse wave velocity, after adjusting for age, sex, body mass index, heart rate, and mean arterial pressure [31]. However, that study employed cf-PWV and heart-femoral pulse wave velocity as a measure of aortic stiffness. These are time-consuming and costly examinations [32], as well as being inconvenient for the purposes of screening.

The ba-PWV value reflects the stiffness of peripheral muscular arteries and is simpler and more convenient to perform [14]. In addition, ba-PWV has been demonstrated to correlate well with cf-PWV, and both have similar predictive power for cardiovascular disease and clinical events [32–34]. However, the association between peripheral arterial stiffness and changes in eGFR in patients with type 2 diabetes remains largely unexplored. Chen et al. [22] reported that ba-PWV was associated with renal function decline and with progression to dialysis or death in both diabetic and nondiabetic patients who had moderate to severe CKD. A recent Japanese study revealed that arterial stiffness, as measured by ba-PWV, could predict the decline of renal function in healthy subjects [35]. However, the latter study did not include diabetic patients and aimed to predict moderate to severe CKD as the endpoint of the study was development of a GFR <60 mL·min^−1^per 1.73 m^2^during a 5-6-year follow-up period [35]. Our study fills this gap and confirms the independent association between ba-PWV and a rapid decline in eGFR in type 2 diabetic patients without apparent cardiovascular disease. Although it is suggested that eGFR declines slowly in most patients with early stage CKD, the rate of eGFR decline varies among individuals, and many factors may impact progression [36]. Because that early CKD progress almost asymptomatically, it needs some laboratory examinations to monitor early CKD. Accordingly, the results of our study, that ba-PWV may serve as a simple and noninvasive examination to detect a rapid renal function progression in type 2 diabetic patients with early CKD, may further help to modify treatment to limit damage and then even improve the clinical outcome. In present study, higher baseline UACR was associated with a more rapid decline of eGFR. In addition, higher baseline baPWV levels were associated with progression of UACR with marginally significant, suggesting that UACR might play certain role in those links. However, only 37% of our studied population collected their single sport urine for UACR measurement. Concerns of relatively small sample size as well as lack of repeated collection were also raised.

Previous studies reported that peripheral arterial occlusive disease, measured by ABI or TBI, is more prevalent in diabetic patients with CKD [10, 11, 37–39]. In the present study, the TBI values were not significantly lower in patients with a rapid decline in renal function possibly due to the short duration of observation, the small sample size, and the exclusion of patients with abnormal baseline ABI values. Hypertension is a well-known, major risk factor for both cardiovascular events and CKD [4, 40, 41]. In addition to SBP and DBP, PP represents the amplitude of the pressure wave signal, and higher PP values were reported in patients who have cardiovascular risk factors [30]. A previous observational study reported that 24-hour ambulatory PP and impaired nocturnal DBP decline were independent predictors of proteinuria progression among patients with type 2 diabetes during an average of 9.5-year follow-up [42]. However, the relationship between PP and eGFR in diabetic patients is not well understood. Our study found that both SBP and PP were independent predictors of rapid renal function decline, a finding that mandates further investigation.

The ba-PWV values obtained from our cohort with type 2 diabetes were higher than those reported previously in studies conducted in healthy populations [43] and similar to those reported in cohorts with cardiovascular disease [44]. These findings suggest that our type 2 diabetic outpatients, even with normal values of ABI, may have had some degree of peripheral arterial stiffness, as measured by ba-PWV.

### 4.2. Traditional Risk Factors for the Progression of CKD

A recent study that followed patients with type 2 diabetes for 10 years reported that the predictors of a decline in eGFR included hypertension, increased HbA1c levels, and insulin use [45]. Previous studies indicated that postprandial hyperglycemia, not fasting hyperglycemia, is a pathophysiological state contributing to vascular failure and may predispose the progression of atherosclerosis [46]. Our data showed that HbA1c level, rather than fasting plasma glucose level, was significantly correlated with continuous eGFR progression, suggesting a potential detrimental effect of elevated postprandial hyperglycemia. Several studies have reported that dyslipidemia and obesity are associated with CKD progression in patients with and without diabetes [9, 47]. However, this association was not observed in the present study perhaps because of the shorter duration of follow-up or the exclusion of patients with a history of severe cardiovascular events and those with abnormal ABI (ABI > 1.3 and ABI < 0.95). A previous study reported that, compared with metformin, sulfonylureas increased the risk of a decline in eGFR, end-stage renal disease, and mortality [48]. We observed a significantly higher percentage of sulfonylurea treatment among those with a rapid decline in eGFR, while the effect was independent of the HbA1C level. The use of statins and certain antihypertensive drugs such as angiotensin-converting enzyme inhibitors or angiotensin receptor blockers was reported to be associated with a slower progression of renal function and reduced cardiovascular risk in patients with PAD [49, 50]. We did not observe significant differences in the use of these medications between the 2 groups.

### 4.3. Limitations

The main limitation of our study is the retrospective nature of the study design. The ba-PWV was only measured once (at enrollment); however, the validation of these methods has been described previously [18]. This study was a hospital-based study; thus, the external validity is limited. However, the demographic characteristics of our patients were similar to the 2009 national population-based epidemiological data in Taiwan relating to patients with type 2 diabetes according to the National Health Insurance database [51]. Moreover, we obtained data on patient medication use only at baseline and any effects of changes in medication uses on eGFR are unknown. Furthermore, patients with a low ABI (<0.95) were excluded. As a result, our findings may not apply to patients with documented peripheral artery occlusive disease. Last, eGFR was estimated by the Modification of Diet in Renal Disease Study equation, and ethnic differences may exist, which would affect the results obtained from this equation, yet the suggested ethnicity correction only applies to those of African or Caribbean descent [19, 52].

## 5. Conclusions

In conclusion, our results demonstrated that, in addition to traditional and vascular functional markers such as HbA1c, SBP, and PP, values of ba-PWV were independently associated with rapid renal function decline in a group of type 2 diabetic patients without symptomatic cardiovascular disease. Screening type 2 diabetic patients using ba-PWV, a simple and noninvasive marker, may help identify patients at high risk for a short-term decline in renal function.

## Figures and Tables

**Table 1:** Baseline characteristics of patients with a rapid decline in eGFR versus those without rapid decline in eGFR.

	Patients without rapid decline in eGFR (*n* = 461)	Patients with rapid decline in eGFR (*n* = 116)	*P* value
Parameter			
Age (years)	63 ± 12	64 ± 10	0.262
Gender: *n* (%)			1.00
Men	235 (51.0%)	59 (50.9%)	
Women	226 (49.0%)	57 (49.1%)	
BMI (kg/m^2^)	25.1 ± 3.5	24.5 ± 3.3	0.126
SBP (mmHg)	141 ± 19	147 ± 21	0.008*
DBP (mmHg)	81 ± 11	82 ± 10	0.433
PP (mmHg)	60 ± 13	64 ± 16	0.002*
Smoking (%)	76 (16.5%)	19 (16.4%)	1.000
ABI	1.11 ± 0.07	1.12 ± 0.07	0.264
TBI	0.79 ± 0.12	0.76 ± 0.13	0.079
ba-PWV (m/s)	18.40 ± 4.16	19.72 ± 4.51	0.003*
FPG (mg/dL)	159 ± 64	161 ± 57	0.686
HbA1c (%)	7.9 ± 1.8	8.5 ± 2.1	0.006*
TC (mg/dL)	186 ± 35	187 ± 50	0.676
TG (mg/dL)	145 ± 111	156 ± 156	0.283
HDL (mg/dL)	43 ± 13	43 ± 14	0.805
LDL (mg/dL)	110 ± 30	108 ± 39	0.552
Cr (mg/dL)	0.87 ± 0.35	0.89 ± 0.43	0.564
eGFR (mLmin^−1^per 1.73 m^2^)	91 ± 27	92 ± 32	0.700
Medicines			
SU	300 (65.1%)	92 (79.3%)	0.004*
Metformin	361 (78.3%)	98 (84.5%)	0.158
TZD	72 (15.7%)	17 (14.7%)	0.886
Insulin	47 (10.2%)	14 (12.1%)	0.612
ACEI/ARB	185 (40.1%)	58 (50.0%)	0.059
Diuretics	34 (7.4%)	12 (10.3%)	0.336
Statin	173 (37.6%)	45 (38.8%)	0.831

BMI: body mass index (kg/m^2^); SBP: systolic blood pressure (mmHg); DBP: diastolic blood pressure (mmHg); PP: pulse pressure (mmHg); ABI: Ankle-brachial index; TBI: toe-brachial index; FPG: fasting plasma glucose (mg/dL); HbA1c: glycosylated hemoglobin (%); TC: total cholesterol (mg/dL); TG: triglyceride (mg/dL); HDL: high-density lipoprotein (mg/dL); LDL: low-density lipoprotein (mg/dL); Cr: creatinine (mg/dL); eGFR: estimated glomerular filtration rate (mLmin^−1^per 1.73 m^2^); ba-PWV: brachial-ankle pulse wave velocity (m/s); SU: sulfonylurea; TZD: thiazolidinedione; ACEI: angiotensin-converting enzyme inhibitor; ARB: angiotensin receptor blocker. The Chi-square test was used to assess differences based on gender, smoking status, and the use of medications; *P < 0.05.

**Table 2:** Unadjusted and age-adjusted odds ratios (95% confidence intervals) of rapid decline in eGFR.

	Unadjusted OR	95% CI		*P* value	Age-adjusted OR	95% CI		*P* value
Parameter								
Age (years)	1.011	0.992	1.029	0.261	—	—	—	—
Gender	0.995	0.662	1.496	0.982	1.023	0.679	1.543	0.912
BMI (kg/m^2^)	0.953	0.896	1.014	0.126	0.958	0.900	1.020	0.181
SBP (mmHg)	1.014	1.004	1.024	0.008*	1.013	1.003	1.024	0.015*
DBP (mmHg)	1.008	0.989	1.027	0.432	1.009	0.989	1.028	0.380
PP (mmHg)	1.022	1.008	1.037	0.002*	1.022	1.007	1.038	0.004*
Smoking (%)	0.992	0.573	1.720	0.978	1.092	0.615	1.937	0.765
ABI	5.241	0.288	95.530	0.263	4.695	0.258	85.552	0.296
TBI	0.240	0.049	1.182	0.079	0.283	0.053	1.505	0.139
ba-PWV (m/s)	1.071	1.023	1.121	0.003*	1.085	1.025	1.149	0.005*
FPG (mg/dL)	1.001	0.997	1.004	0.685	1.001	0.998	1.004	0.539
HbA1c (%)	1.145	1.039	1.262	0.006*	1.163	1.053	1.284	0.003*
TC (mg/dL)	1.001	0.996	1.006	0.675	1.002	0.996	1.007	0.513
TG (mg/dL)	1.001	0.999	1.002	0.296	1.001	0.999	1.002	0.202
HDL (mg/dL)	0.998	0.982	1.014	0.804	0.997	0.982	1.013	0.753
LDL (mg/dL)	0.998	0.992	1.004	0.551	0.999	0.992	1.005	0.673
eGFR (mLmin^−1^per 1.73 m^2^)	1.001	0.994	1.009	0.700	1.004	0.996	1.012	0.327
Medicines								
SU	2.057	1.262	3.525	0.004	2.135	1.305	3.493	0.003
Metformin	1.508	0.871	2.612	0.143	1.577	0.906	2.744	0.107
TZD	0.925	0.522	1.641	0.791	0.963	0.541	1.717	0.899
Insulin	1.206	0.639	2.276	0.563	1.239	0.655	2.343	0.510
ACEI/ARB	1.492	0.991	2.245	0.055	1.454	0.962	2.197	0.076
Diuretics	0.690	0.345	1.379	0.296	0.732	0.363	1.479	0.385
Statin	1.051	0.692	1.598	0.814	1.055	0.694	1.603	0.804

BMI: body mass index (kg/m^2^); SBP: systolic blood pressure (mmHg); DBP: diastolic blood pressure (mmHg); PP: pulse pressure (mmHg); ABI: ankle-brachial index; TBI: toe-brachial index; FPG: fasting plasma glucose (mg/dL); HbA1c: glycosylated hemoglobin (%); TC: total cholesterol (mg/dL); TG: triglyceride (mg/dL); HDL: high-density lipoprotein (mg/dL); LDL: low-density lipoprotein (mg/dL); Cr: creatinine (mg/dL); eGFR: estimated glomerular filtration rate (mLmin^−1^per 1.73 m^2^); ba-PWV: brachial-ankle pulse wave velocity (m/s); SU: sulfonylurea; TZD: thiazolidinedione; ACEI: angiotensin-converting enzyme inhibitor; ARB: angiotensin receptor blocker. The annual decline of eGFR appears to be a part of the normal physiologic process of organ senescence; we evaluated the potential risk factors after adjusting for the effect of age; OR: Odds ratio; 95% CI: 95% confidence interval; *P < 0.05.

**Table 3:** Pearson correlation coefficients between the change of eGFR values and markers of peripheral arterial function.

	Baseline eGFR	12-month eGFR	^§^Delta eGFR	^¶^eGFR change rate	ba-PWV	SBP	PP	TBI
Baseline eGFR	1							
12-month eGFR	0.788**	1						
Delta eGFR	−0.217**	0.430**	1					
eGFR change rate	−0.095*	0.492**	0.917**	1				
ba-PWV	−0.260**	−0.296**	−0.087*	−0.099*	1			
SBP	−0.130**	−0.178**	−0.091*	−0.132**	0.524**	1		
PP	−0.149**	−0.189**	−0.080	−0.122**	0.544**	0.853**	1	
TBI	0.196**	0.231**	0.078	0.104*	−0.243**	−0.178**	−0.290**	1

^§^Delta eGFR (mLmin^−1^per 1.73 m^2^) = 12-month eGFR − baseline eGFR = annual eGFR change.

^¶^eGFR change rate (%) = 12-month eGFR − baseline eGFR/baseline eGFR = annual eGFR change rate (%).

ba-PWV: brachial-ankle pulse wave velocity (m/s); SBP: systolic blood pressure (mmHg); PP: pulse pressure (mmHg); TBI: toe-brachial index; **P < 0.01; *P < 0.05; *n* = 577.

**Table:** (a) Multivariate logistic regression models were built by adding ba-PWV, PP, SBP, and TBI separately after adjusting for potential confounders, including gender, age, body mass index, smoking, HbA1c, and baseline eGFR

	Model 1				Model 2				Model 3				Model 4			
	OR	95% CI		*P*	OR	95% CI		*P*	OR	95% CI		*P*	OR	95% CI		*P*
Age	0.999	0.972	1.026	0.923	1.010	0.987	1.033	0.402	1.004	0.980	1.028	0.763	1.013	0.990	1.036	0.282
Sex	1.050	0.671	1.642	0.832	1.094	0.696	1.718	0.697	1.223	0.768	1.948	0.397	1.036	0.664	1.616	0.876
BMI	0.969	0.909	1.033	0.331	0.955	0.895	1.019	0.165	0.957	0.897	1.020	0.178	0.969	0.909	1.033	0.341
Smoking	0.992	0.529	1.861	0.980	0.995	0.530	1.867	0.987	1.000	0.531	1.883	1.000	0.999	0.534	1.868	0.998
HbA1c	1.133	1.022	1.256	0.017	1.150	1.039	1.273	0.007	1.150	1.038	1.273	0.007	1.149	1.039	1.271	0.007
eGFR	1.002	0.994	1.010	0.617	1.002	0.994	1.010	0.564	1.002	0.994	1.010	0.644	1.002	0.994	1.011	0.562
ba-PWV	1.072	1.011	1.136	0.020												
SBP					1.014	1.004	1.025	0.009								
PP									1.025	1.008	1.041	0.003				
TBI													0.338	0.062	1.846	0.210

**Table:** (b) Multivariate logistic regression models that were adjusted for the confounders as in, Table 4(a) and furthermore, we also adjusted the potential influence of sulfonylurea use (independent-sample *t*-test *P* = 0.004), and the table shows that markers of arterial stiffness remained independent risk factors for a rapid decline in eGFR, whereas the effects of HbA1C on rapid renal function progression became nonsignificant

	Model 1				Model 2				Model 3				Model 4			
	OR	95% CI		*P*	OR	95% CI		*P*	OR	95% CI		*P*	OR	95% CI		*P*
Sex	1.000	0.974	1.028	0.987	1.010	0.987	1.034	0.394	1.004	0.980	1.029	0.739	1.013	0.990	1.037	0.265
Age	1.047	0.667	1.643	0.843	1.093	0.693	1.723	0.703	1.212	0.758	1.938	0.421	1.033	0.660	1.617	0.887
BMI	0.964	0.904	1.028	0.270	0.951	0.890	1.015	0.130	0.953	0.893	1.017	0.145	0.965	0.905	1.029	0.281
Smoking	1.016	0.539	1.913	0.961	1.023	0.543	1.926	0.945	1.025	0.543	1.936	0.939	1.023	0.545	1.920	0.944
HbA1c	1.097	0.984	1.222	0.095	1.108	0.995	1.234	0.061	1.110	0.996	1.237	0.059	1.110	0.997	1.235	0.057
eGFR	1.001	0.993	1.009	0.757	1.002	0.994	1.010	0.693	1.001	0.993	1.009	0.775	1.002	0.993	1.010	0.717
SU	1.773	1.056	2.977	0.030	1.862	1.108	3.128	0.019	1.802	1.072	3.029	0.026	1.819	1.085	3.049	0.023
ba-PWV	1.066	1.005	1.130	0.033												
SBP					1.014	1.004	1.025	0.009								
PP									1.024	1.008	1.041	0.004				
TBI													0.382	0.070	2.092	0.267

OR: odds ratio; 95% CI: 95% confidence interval.
